# Antiproliferation- and Apoptosis-Inducible Effects of a Novel Nitrated [6,6,6]Tricycle Derivative (SK2) on Oral Cancer Cells

**DOI:** 10.3390/molecules27051576

**Published:** 2022-02-27

**Authors:** Sheng-Chieh Wang, Meng-Yang Chang, Jun-Ping Shiau, Ammad Ahmad Farooqi, Yu-Hsiang Huang, Jen-Yang Tang, Hsueh-Wei Chang

**Affiliations:** 1 Department of Biomedical Science and Environmental Biology, Ph.D. Program in Life Sciences, College of Life Sciences, Kaohsiung Medical University, Kaohsiung 80708, Taiwan; u107851101@gap.kmu.edu.tw; 2 Department of Medicinal and Applied Chemistry, Kaohsiung Medical University, Kaohsiung 80708, Taiwan; mychang@kmu.edu.tw; 3 Department of Surgery, Kaohsiung Medical University Hospital, Kaohsiung 80708, Taiwan; drshiaoclinic@gmail.com; 4 Division of Breast Surgery and Department of Surgery, Kaohsiung Medical University Hospital, Kaohsiung 80708, Taiwan; 5 Institute of Biomedical and Genetic Engineering (IBGE), Islamabad 54000, Pakistan; farooqiammadahmad@gmail.com; 6 Post-Graduate Year Training (PGY), Department of Clinical Education and Training, Kaohsiung Medical University Hospital, Kaohsiung 80708, Taiwan; johnhuang851205@gmail.com; 7 School of Post-Baccalaureate Medicine, Kaohsiung Medical University, Kaohsiung 80708, Taiwan; 8 Department of Radiation Oncology, Kaohsiung Medical University Hospital, Kaoshiung Medical University, Kaohsiung 80708, Taiwan; 9 Institute of Medical Science and Technology, National Sun Yat-sen University, Kaohsiung 80424, Taiwan; 10 Center for Cancer Research, Kaohsiung Medical University, Kaohsiung 80708, Taiwan

**Keywords:** nitrated [6,6,6]tricycles, apoptosis, DNA damage, antiproliferation, oral cancer

## Abstract

The benzo-fused dioxabicyclo[3.3.1]nonane core is the central framework in several natural products. Using this core, we had developed a novel nitrated [6,6,6]tricycle-derived compound containing an *n*-butyloxy group, namely, SK2. The anticancer potential of SK2 was not assessed. This study aimed to determine the antiproliferative function and investigated possible mechanisms of SK2 acting on oral cancer cells. SK2 preferentially killed oral cancer cells but caused no harmful effect on non-malignant oral cells. After the SK2 exposure of oral cancer cells, cells in the sub-G1 phase accumulated. This apoptosis-like outcome of SK2 treatment was validated to be apoptosis via observing an increasing annexin V population. Mechanistically, apoptosis signalers such as pancaspase, caspases 8, caspase 9, and caspase 3 were activated by SK2 in oral cancer cells. SK2 induced oxidative-stress-associated changes. Furthermore, SK2 caused DNA damage (γH2AX and 8-hydroxy-2′-deoxyguanosine). In conclusion, a novel nitrated [6,6,6]tricycle-derived compound, SK2, exhibits a preferential antiproliferative effect on oral cancer cells, accompanied by apoptosis, oxidative stress, and DNA damage.

## 1. Introduction

Oral cancer is a malignancy that develops in the lips, mouth, or throat tissues. In Taiwan, oral cancer represents the third most common malignancy and is the fourth leading cause of death due to cancer in males [1]. Oral cancer also occurs globally [2]. Due to diagnosis at advanced stages, the five-year survival rate for oral cancer is low [3], giving prominence to the benefit of refining our understanding of the pathogenesis of human oral carcinogenesis [4]. In addition to surgery, chemo- and radiotherapy are alternative ways for curing oral cancer, but they commonly generate severe side effects [5]. Therefore, anticancer drugs with low side effects can improve the effectiveness of oral cancer therapy.

The dioxabicyclo[3.3.1]nonane core exists in several natural products and chemical drugs. For example, 1,3-disubstituted 2,9-dioxabicyclo[3.3.1]nonane is the core of rings F/G in the marine algal toxins azaspiracids [6]. The 2,8-dioxabicyclo[3.3.1]nonane core can be used for anticoagulants [7]. Natural products, such as epicoccolide A [8] and epicoconigrone A [9], exhibit the benzo-fused dioxabicyclo[3.3.1]nonane core as the central framework. Epicoccolide A shows antifungal activity [10]. However, the anticancer effects of dioxabicyclo[3.3.1]nonane derivatives remain unclear.

Using this benzo-fused dioxabicyclo[3.3.1]nonane core, we had developed a novel nitrated [6,6,6]tricycle-derived compound containing an *n*-butyloxy group, namely, SK2 [11]. SK2 possesses an N-O bond similar to the O-O bond, which is prone to chemical cleavage [12] and forms a free-radical structure. Moreover, SK2 possesses a NO_2_ group that is a radical initiator or promoter [13]. Accordingly, the N-O bond and NO_2_ group in SK2 may generate free radicals. Therefore, we expect that it may potentially modulate oxidative or nitrative stresses. The nitration-mediated inhibition of antioxidant enzymes, such as manganese superoxide dismutase, enhances superoxide levels and oxidative/nitrative stress [14], causing DNA damage [15], autophagy, and apoptosis [16,17]. This warrants the detailed investigation of the potential anticancer effect of SK2.

The purpose of the present study was to assess the antiproliferative functions and mechanisms of SK2 acting on oral cancer cells by measuring proliferation, the cell cycle, cellular and mitochondrial oxidative stress, apoptosis, and the DNA-damage status.

## 2. Results

### 2.1. SK2 Preferentially Kills Oral Cancer Cells

**Figure 1:**
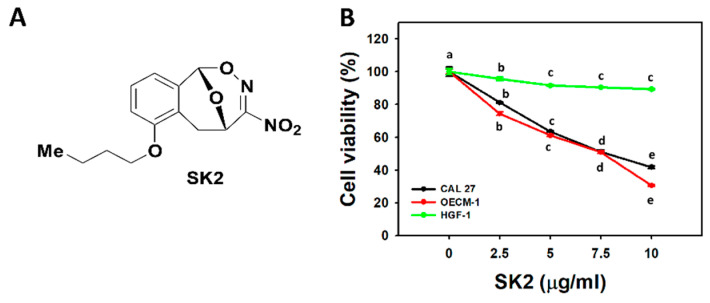
Structure and cell viability effect of SK2. (**A**) Structures of SK2. IUPAC name: 6-*n*-butoxy-10-nitro-12,13-dioxa-11-azatricyclo[7.3.1.0^2,7^]trideca-2,4,6,10-tetraene. (**B**) Cell viability. Oral cancer cells (CAL 27 and OECM-1) and non-malignant oral cells (HGF-1) were exposed to SK2 (0 (0.1% dimethyl sulfoxide (DMSO)), 2.5, 5, 7.5, and 10 µg/mL; 0, 8.56, 17.11, 25.68, and 34.23 µM) for 24 h, and their viabilities were assessed by MTS assays. Data (means ± SDs; *n* = 3) without overlapping characters differed significantly (*p* < 0.0001). For an example of CAL 27 and OECM-1 cells, different concentrations showing different characters indicate significantly different results. For HGF-1 cells, for SK2 at 0, 2.5, and 5 µg/mL, “a, b, and c” indicate significant differences. For HGF-1 cells, for SK2 at 5, 7.5, and 10 µg/mL, the same letter “c” indicates non-significant differences because the results overlapped.

The structure of SK2 is provided (Figure 1A). The cell viability was dose-dependently inhibited by SK2 treatments of oral cancer cells (CAL 27 and OECM-1). Moreover, the cytotoxicity for non-malignant oral cells was examined. SK2 showed a minor cytotoxic effect (>90% viability) on non-malignant oral cells (HGF-1) (Figure 1B). These results reveal that SK2 exerts preferential antiproliferative effects on oral cancer cells but does not affect non-malignant oral cells.

### 2.2. SK2 Disturbs Cell Cycle Progression

**Figure 2:**
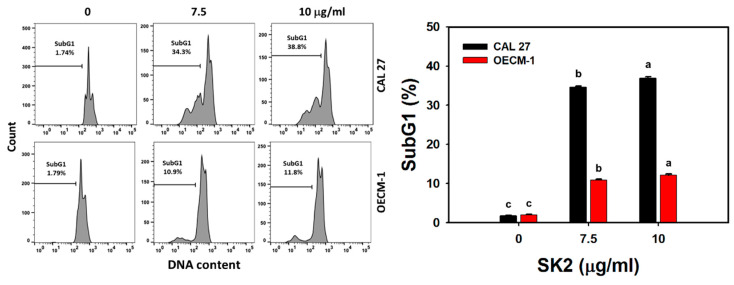
SK2 causes sub-G1 accumulation in oral cancer cells. DNA-content detections and statistics are provided. Cells were exposed to SK2 (0 (0.1% DMSO), 7.5, and 10 µg/mL) for 24 h. Data (means ± SDs; *n* = 3) without overlapping characters differed significantly (*p* < 0.05). For an example of CAL 27 and OECM-1 cells, different concentrations with different characters produced significantly different results. For CAL 27 and OECM-1 cells, for SK2 at 0, 7.5, and 10 µg/mL, “c, b, and a” indicate significant differences because the results did not overlap.

After the SK2 treatment of oral cancer cells, the cell cycle distribution was assessed (Figure 2). The SK2 treatments dose-dependently induced the accumulation of the sub-G1 population of oral cancer cells.

### 2.3. SK2 Increases Annexin V/7AAD-Assessed Apoptosis

**Figure 3:**
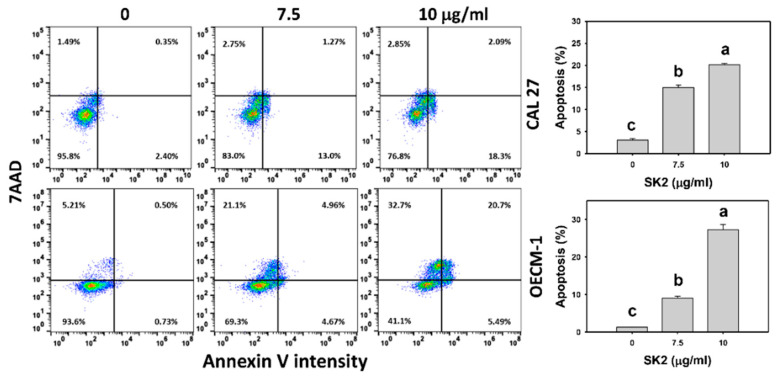
SK2 triggers apoptosis in oral cancer cells. Annexin V/7AAD detections and statistics are provided. Cells were exposed to SK2 (0 (0.1% DMSO), 7.5, and 10 µg/mL) for 24 h. Annexin V(+)/7AAD (+ or −) was regarded as the apoptosis %. Data (means ± SDs; *n* = 3) without overlapping characters differed significantly (*p* < 0.05). For an example of CAL 27 and OECM-1 cells, different concentrations with different characters produced significantly different results. For CAL 27 and OECM-1 cells, for SK2 at 0, 7.5, and 10 µg/mL, “c, b, and a” indicate significant differences because the data did not overlap.

After the SK2 treatment of oral cancer cells, the distribution of annexin V/7AAD was assessed (Figure 3). The SK2 treatments dose-dependently induced the annexin V-positive (+) population of oral cancer cells.

### 2.4. SK2 Increases Caspase-Signaling Activation

**Figure 4:**
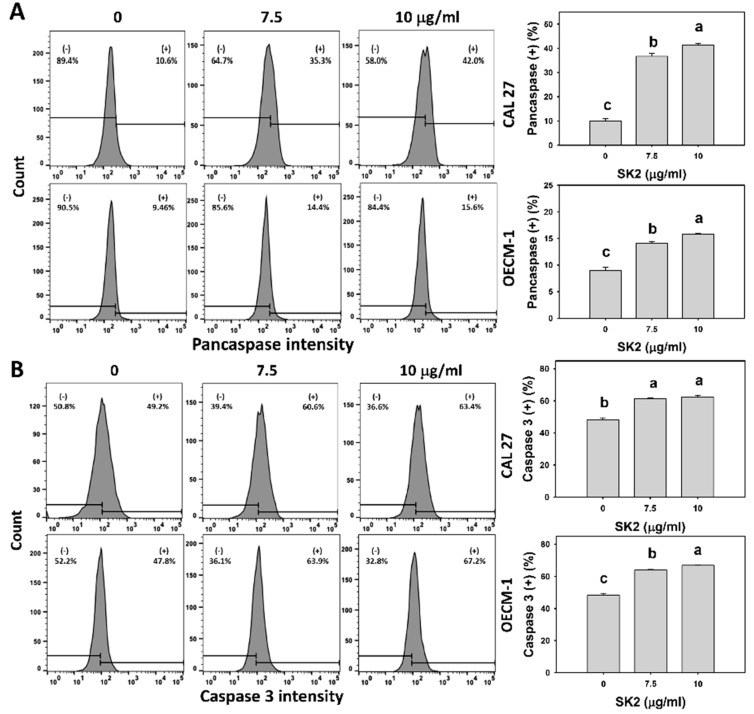

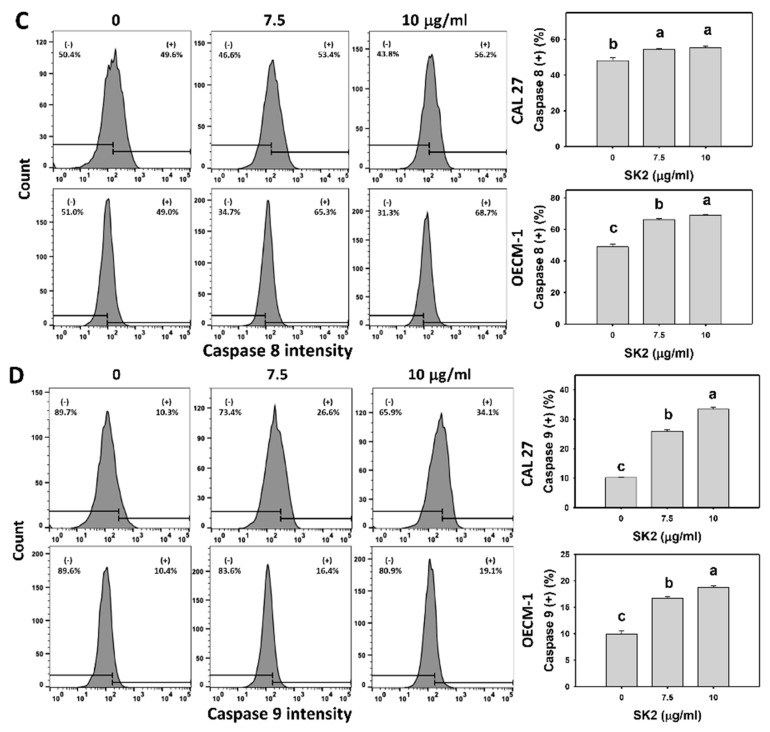
SK2 triggers caspase signaling in oral cancer cells. (**A**–**D**) Pancaspase and Cas 3, 8, and 9 detections, and statistics are provided. Cells were exposed to SK2 (0 (0.1% DMSO), 7.5, and 10 µg/mL) for 24 h. (+) shown in each panel represents pancaspase and Cas 3, 8, and 9 (+). Data (means ± SDs; *n* = 3) without overlapping characters differed significantly (*p* < 0.05). For CAL 27 and OECM-1 cells (**D**), for SK2 at 0, 7.5, and 10 µg/mL, “c, b, and a” indicate significant differences because the data did not overlap. For an example of CAL 27 cells (**B**,**C**), for SK2 at 7.5 and 10 µg/mL, the same letter “a” indicates non-significant differences because the data overlapped.

Apoptosis is initiated and promoted by apoptotic signaling such as caspases [18]. After the SK2 treatment of oral cancer cells, the pancaspase expression was assessed (Figure 4A). Based on flow cytometry analysis, the pancaspase-positive (+) population, i.e., pancaspase activation, of oral cancer cells was dose-dependently induced by SK2 treatments.

Since pancaspase only enables detection for general caspases such as caspase-1 and 3 to 9 [19], specific caspases such as the extrinsic, intrinsic, and executor caspases (Cas 8, 9, and 3) were further examined (Figure 4B–D). Based on flow cytometry analysis, the Cas 8, 9, and 3 (+) populations were dose-dependently induced by SK2 treatments.

### 2.5. SK2 Increases ROS Induction

**Figure 5:**
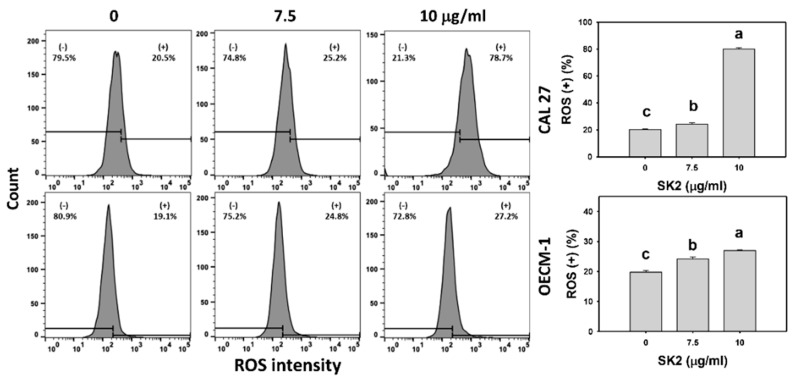
SK2 prompts ROS induction in oral cancer cells. ROS detections and statistics are provided. Cells were exposed to SK2 (0 (0.1% DMSO), 7.5, and 10 µg/mL) for 24 h. (+) in each panel represents ROS (+). Data (means ± SDs; *n* = 3) without overlapping characters differed significantly (*p* < 0.05). For an example of CAL 27 and OECM-1 cells, different concentrations with different characters produced significantly different results. For CAL 27 and OECM-1 cells, for SK2 at 0, 7.5, and 10 µg/mL, “c, b, and a” indicate significant differences because the data did not overlap.

The oxidative-stress status of the oral cancer cells was assessed by the ROS level. After the SK2 treatment of oral cancer cells, the ROS levels were assessed (Figure 5). Based on flow cytometry analysis, the ROS-positive (+) population of oral cancer cells was dose-dependently induced by SK2 treatments.

### 2.6. SK2 Increases Mitochondrial Superoxide (MitoSOX) Induction

**Figure 6:**
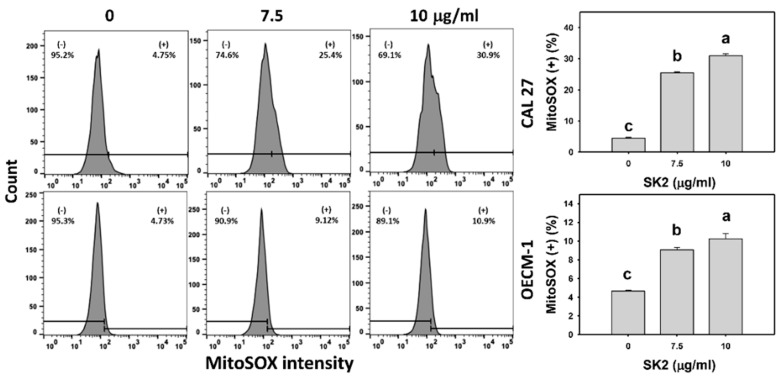
SK2 prompts MitoSOX induction in oral cancer cells. MitoSOX detections and statistics are provided. Cells were exposed to SK2 (0 (0.1% DMSO), 7.5, and 10 µg/mL) for 24 h. (+) in each panel represents MitoSOX (+). Data (means ± SDs; *n* = 3) without overlapping characters differed significantly (*p* < 0.05). For an example of CAL 27 and OECM-1 cells, different concentrations with different characters produced significantly different results. For CAL 27 and OECM-1 cells, for SK2 at 0, 7.5, and 10 µg/mL, “c, b, and a” indicate significant differences because the data did not overlap.

The oxidative-stress status of oral cancer cells was also assessed by the MitoSOX level. After the SK2 treatment of oral cancer cells, the MitoSOX levels were assessed (Figure 6). The MitoSOX-positive (+) population of oral cancer cells was dose-dependently induced by SK2 treatments based on flow cytometry analysis.

### 2.7. SK2 Increases Mitochondrial Membrane Potential (MMP) Destruction

**Figure 7:**
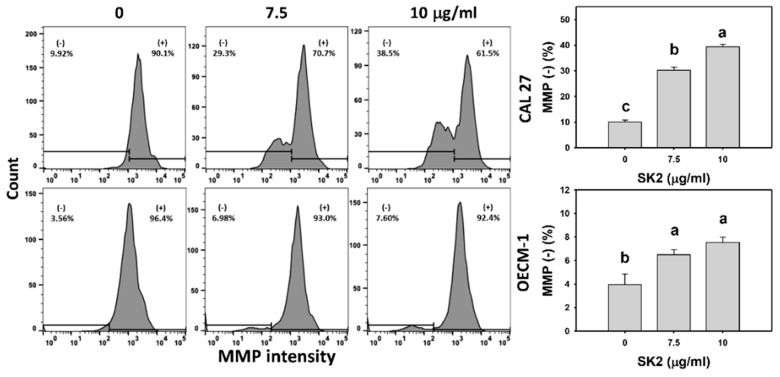
SK2 prompts MMP destruction in oral cancer cells. MMP detections and statistics are provided. Cells were exposed to SK2 (0 (0.1% DMSO), 7.5, and 10 µg/mL) for 24 h. (−) in each panel represents MMP (−). Data (means ± SDs; *n* = 3) without overlapping characters differed significantly (*p* < 0.05). For an example of CAL 27 cells, different concentrations with different characters indicate significant differences. For an example of OECM-1 cells, for SK2 at 7.5 and 10 µg/mL, the same letter “a” indicates non-significant differences because the results overlapped. For CAL 27 cells, for SK2 at 0, 7.5, and 10 µg/mL, “c, b, and a” indicate significant differences because the results did not overlap. For OECM-1 cells, for SK2 at 7.5 and 10 µg/mL, “a and a” indicate the results are not significantly different because they overlapped.

The oxidative-stress status of oral cancer cells was further measured by the MMP level. After the SK2 treatment of oral cancer cells, the MMP levels were assessed (Figure 7). Based on flow cytometry analysis, the MMP-negative (−) population of oral cancer cells was induced by SK2 treatments.

### 2.8. SK2 Increases γH2AX Induction

**Figure 8:**
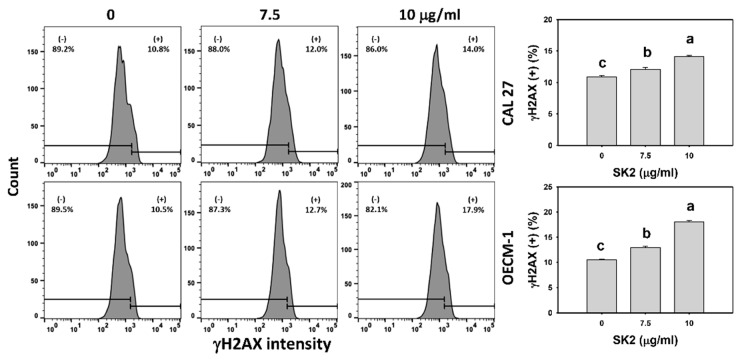
SK2 prompts γH2AX induction in oral cancer cells. γH2AX detections and statistics are provided. Cells were exposed to SK2 (0 (0.1% DMSO), 7.5, and 10 µg/mL) for 24 h. (+) in each panel represents γH2AX (+). Data (means ± SDs; *n* = 3) without overlapping characters differed significantly (*p* < 0.05). For an example of CAL 27 and OECM-1 cells, for different concentrations, different characters indicate significant differences.

The DNA damage of oral cancer cells was assessed by the γH2AX level, a DNA-double-strand-break biomarker [20]. After the SK2 treatment of oral cancer cells, the γH2AX levels were assessed (Figure 8). The γH2AX-positive (+) population of oral cancer cells was dose-dependently induced by SK2 treatments based on flow cytometry analysis.

### 2.9. SK2 Increases 8-Hydroxy-2′-deoxyguanosine (8-OHdG) Induction

**Figure 9:**
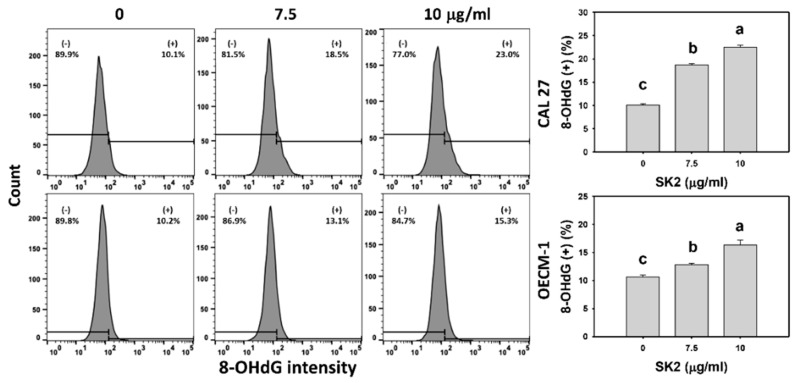
SK2 prompts 8-OHdG induction in oral cancer cells. 8-OHdG detections and statistics are provided. Cells were exposed to SK2 (0 (0.1% DMSO), 7.5, and 10 µg/mL) for 24 h. (+) in each panel represents 8-OHdG (+). Data (means ± SDs; *n* = 3) without overlapping characters differed significantly (*p* < 0.05). For an example of CAL 27 and OECM-1 cells, for different concentrations, different characters indicate significant differences.

The DNA-damage status of oral cancer cells was assessed by the 8-OHdG level [21]. After the SK2 treatment of oral cancer cells, the 8-OHdG levels were assessed (Figure 9). The 8-OHdG-positive (+) population of oral cancer cells was dose-dependently induced by SK2 treatments based on flow cytometry analysis.

## 3. Discussion

The present study examined the proliferation-modulating effects and investigated the anticancer mechanisms of SK2 in oral cancer cells. The oxidative-stress generation, apoptosis signaling, and DNA-damage induction were assessed in oral cancer cells following SK2 treatment.

The IC_50_ values for several clinical drugs for oral cancer cells are reported for comparison. For cisplatin, the IC_50_ values were 5.3 and 5 μM for oral cancer cells (CAL 27) based on the 24 h MTT [22] and MTS [23] methods. For curcumin, the IC_50_ was 12 μM for CAL 27 cells based on the 24 h sulforhodamine B method [24]. For 5-fluorouracil, the IC_50_ was 95.6 μM for CAL 27 cells based on the 24 h Alamar blue method [25]. Moreover, cisplatin [26], curcumin [27], and 5-fluorouracil [28] showed side effects.

Although dioxabicyclo[3.3.1]nonane derivatives have frequently been synthesized, their anticancer effects have rarely been investigated. Recently, peniciketal A, a *Penicillium raistrickii*-derived spiroketal compound containing a benzo-fused 2,8-dioxabicyclo[3.3.1]nonane core, was discussed [29,30]. Peniciketal A had IC_50_ values of 33.50, 56.85, and 60.64 μM for the 24 h treatment and 18.99, 45.14, and 39.94 μM for the 48 h treatment of leukemia cells (THP-1, K562, and HL60). Peniciketal A induced apoptosis in and preferential antiproliferative effects on leukemia cells but not for primary mouse embryonic fibroblasts (MEFs) [29]. A sanctis B-containing dibenzo-2,8-dioxabicyclo[3.3.1]nonane scaffold exhibited antiproliferative effects on breast cancer cells, but no detailed anticancer mechanism was reported [31]. Similarly, the IC_50_ value of SK2 at 24 h of exposure for oral cancer cells (CAL 27 and OECM-1) was 7.5 μg/mL (25.68 μM), but it showed noncytotoxicity to non-malignant oral cells (HGF-1) based on the MTS assay. These results suggest that dioxabicyclo[3.3.1]nonane derivatives have a preferential antiproliferative ability for cancer cells rather than normal cells. This low cytotoxic property for normal cells may potentially contribute to its low side effects. This warrants a detailed examination of the in vivo antitumor effects of SK2 by using an animal model.

The cellular ROS-induction effects of dioxabicyclo[3.3.1]nonane derivatives remain unclear. SK2 contains a N-O bond and NO_2_ group that are competent in forming a free-radical structure [12,13]. Accordingly, oxidative-stress effects of SK2 in oral cancer cells were examined. The present study first reported that SK2 induced cellular and mitochondrial oxidative stress and MMP destruction in oral cancer cells. Several natural products and chemical agents can preferentially kill cancer cells by oxidative-stress induction in cancer cells rather than normal cells [32,33]. The basal ROS level in most cancer cells is generally higher than that in normal cells, making cancer cells unable to tolerate the exogenous ROS generated by ROS-modulating drugs, causing the preferential antiproliferative effects on cancer cells [33]. Accordingly, this rationale may partially explain the preferential antiproliferative effect of SK2 on oral cancer cells rather than non-malignant oral cells.

Several oxidative-stress-generating agents trigger apoptosis [34,35,36,37,38,39,40]. Consistently, SK2 induced apoptosis, with an annexin V increment and Cas 3-cleavage evidence. Moreover, SK2 enhanced the cleavages of Cas 8 and Cas 9, indicating that SK2 triggered intrinsic and extrinsic apoptotic signaling in oral cancer cells. Oxidative-stress-generating agents also enhanced DNA damage [36,37,40]. Similarly, SK2 caused DNA damage as assessed using γH2AX and 8-OHdG markers in oral cancer cells. Accordingly, these results demonstrate that oxidative stress, apoptosis, and DNA damage were involved in the antiproliferative ability of SK2 in oral cancer cells.

A combined treatment approach has benefits for targeting different molecular mechanisms [41,42] for anticancer therapy. For example, the natural product cordycepin combined with the clinical drug cisplatin showed synergistic apoptotic effects on oral cancer cells [43]. A synthesized chemical, sulfonyl chromen-4-ones (CHW09), had synergistic effects of inhibiting oral cancer cell proliferation when combined with either UVC [44] or X-ray [45] irradiation. Since SK2 is noncytotoxic to non-malignant oral cells (HGF-1), the potential synergistic effects of combined treatment with SK2 and other anticancer drugs or radiation warrant a detailed evaluation of oral cancer therapy in the future.

The MetaCore/MetaDrug platform [46,47] uses QSAR models to predict the input molecule’s molecular pathway and pharmacokinetic activity. The potential targets were unavailable after data retrieval on 27 November 2021. Moreover, a similar structure was unavailable for data mining using MetaCore/MetaDrug. Therefore, the potential targets cannot be predicted using this platform. It is noted that ROS are also generated when the antioxidant machinery is overloaded [48,49]. When antioxidant signaling is suppressed, ROS are generally developed. For example, pomegranate extract downregulated several antioxidant signaling pathways, enhancing oxidative stress, in oral cancer cells [50]. Accordingly, SK2 may downregulate the antioxidant signaling pathway, inducing ROS generation. The possibility that antioxidant signaling proteins were the potential targets needs to be further assessed in the future.

## 4. Materials and Methods

### 4.1. SK2 Preparation

SK2 (MW = 292.1059), a nitrated [6.6.6]tricycle-derived compound containing an *n*-butoxy group, was prepared as previously described [11]. A representative synthetic procedure for SK2 is as follows: HNO_3_ (97%, 0.5 mL) was mixed with a solution of 2-allyl-3-*n*-butoxybenzaldehyde (218 mg, 1.0 mmol) in H_2_SO_4_ (98%, 2 mL) at 25 °C. Subsequently, they were stirred at 80 °C for 24 h. After dilution with water (10 mL), they were extracted with CH_2_Cl_2_ (3 × 20 mL). These organic layers were processed by washing with brine, drying, and filtering. They were evaporated to provide a crude product using a reduced pressure machine. Finally, they were purified using silica gel (hexanes/EtOAc = 4/1~1/1) to generate SK2 (213 mg) with a 73% yield. The purity of the SK2 was >95% and was determined from ^1^H- and ^13^C-NMR spectra. The SK2 was dissolved in DMSO (Sigma-Aldrich, St. Louis, MO, USA) for drug treatment.

### 4.2. Cell Cultures and Cell Viability

Oral cancer cell lines (CAL 27) and non-malignant oral cell lines (HGF-1) were obtained from ATCC (Manassas, VA, USA). Another oral cancer cell line, OECM-1 [51], was provided by Dr. Wan-Chi Tsai at Kaohsiung Medical University, Kaohsiung, Taiwan. They were cultured in a standard medium mixed with 10% fetal bovine serum (FBS) and standard antibiotics at 37 °C in a 5% CO_2_ chamber [52]. The cell viability was evaluated using the tetrazolium dye of the MTS reagent (Promega, Madison, WI, USA) [34].

### 4.3. Cell Cycle Assay

The cell cycle phases were determined by their different cellular DNA contents as revealed by probing with 7-aminoactinomycin D (7AAD) (Biotium Inc., Hayward, CA, USA) [53]. The cells were fixed using 75% ethanol overnight. Subsequently, the cells were maintained with 1 μg/mL of 7AAD for 30 min at 37 °C. Finally, flow cytometry analysis was performed using an Accuri C6 flow cytometer (BD Biosciences, Franklin Lakes, NJ, USA) using the FL1 channel.

### 4.4. Annexin V/7AAD Assay

Annexin V can be used to probe phosphatidylserine, regarded as an apoptosis marker. The Annexin V (Strong Biotech Corp., Taipei, Taiwan)/7AAD method [37] was applied. Cells were treated with annexin V-FITC (10 μg/mL) and 7AAD (1 μg/mL, 30 min, 37 °C). Finally, flow cytometry analysis was performed using an Accuri C6 using the FL1 and FL3 channels.

### 4.5. Caspase-Signaling Assay

Caspase-signaling activators, including pancaspase, Cas 3, Cas 8, and Cas 9, were chosen for flow cytometry analysis. A pancaspase-FITC kit (Abcam, Cambridge, UK) enables a general detection of caspase-signaling activators such as caspase-1 and 3 to 9 [19]. Moreover, the individual activity of Cas 3, Cas 8, and Cas 9 was detected using OncoImmunin kits (Gaithersburg, MD, USA) [54,55]. Cells were treated with 10 μM substrate (PhiPhiLux-G1D2, CaspaLux8-L1D2, and CaspaLux9-M1D2) (1:1000) at 37 °C for 1 h. These substrates were individually cleaved by activated Cas 3, Cas 8, and Cas 9 to generate green fluorescence. Finally, flow cytometry analysis was performed using an Accuri C6 using the FL1 channel.

### 4.6. ROS Assay

ROS were probed using 2′,7′-dichlorodihydrofluorescein diacetate (H_2_DCF-DA) (Sigma-Aldrich; St. Louis, MO, USA), producing fluorescence for flow cytometry [38]. Cells were treated with 100 nM H_2_DCF-DA for 30 min. Finally, flow cytometry analysis was performed using an Accuri C6 using the FL1 channel.

### 4.7. MitoSOX Assay

The MitoSOX was probed using MitoSOX™ Red (Molecular Probes, Invitrogen, Eugene, OR, USA), producing fluorescence for flow cytometry [55]. In brief, cells were treated with 5 μM MitoSOX at 37 °C for 30 min. Finally, flow cytometry analysis was performed using an Accuri C6 using the FL3 channel.

### 4.8. MMP Assay

The MMP was probed using MitoProbe™ DiOC_2_(3) (Invitrogen, San Diego, CA, USA), producing fluorescence for flow cytometry [55]. Cells were treated with 20 nM DiOC_2_(3) for 30 min. Finally, flow cytometry analysis was performed using an Accuri C6 using the FL1 channel.

### 4.9. γH2AX Assay

Utilizing flow cytometry, the γH2AX level was detected using a p-Histone H2A.X (Ser 139) antibody (Santa Cruz Biotechnology, Santa Cruz, CA, USA) (1:50 dilution) at 4 °C for 1 h [55]. Subsequently, an Alexa Fluor 488-conjugated secondary antibody (Jackson Laboratory, Bar Harbor, ME, USA) (1:50 dilution) was used for 30 min at RT. Finally, flow cytometry analysis was performed using an Accuri C6 using the FL1 channel.

### 4.10. 8-OHdG Assay

Utilizing flow cytometry, 8-OHdG, an oxidative nucleotide marker, was assessed using an 8-OHdG-FITC antibody (Santa Cruz Biotechnology) at a 100X dilution for 1 h at RT [55]. Finally, flow cytometry analysis was performed using an Accuri C6 using the FL1 channel.

### 4.11. Statistics

The data were multi-compared, and the significance was determined using the JMP 12 software (SAS Institute, Cary, NC, USA) with ANOVA and the Tukey post hoc test. Data without overlapping small characters differed significantly. The data are shown as the means ± SDs for triplicate experiments.

## 5. Conclusions

**Figure 10:**
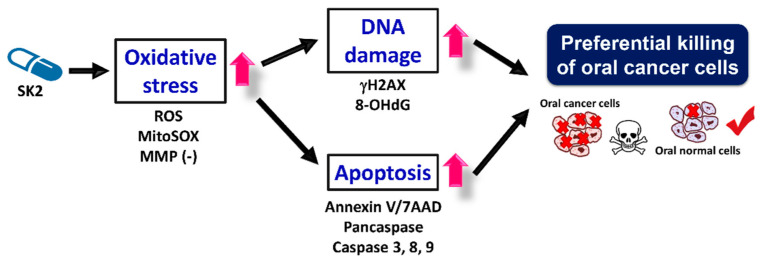
Summary of the mechanism for the anticancer effects of SK2 on oral cancer cells.

We previously used the benzo-fused dioxabicyclo[3.3.1]nonane core to synthesize a novel nitrated [6,6,6]tricycle-derived compound containing an *n*-butyloxy group (SK2). However, the biological function of SK2 was not investigated. The present study validated that SK2 preferentially killed oral cancer cells but showed no harmful effect on non-malignant oral cells. Mechanistically, SK2 induced oxidative stress, triggered apoptosis, and caused DNA damage to oral cancer cells (Figure 10). Elucidating these mechanisms may improve the anticancer application of SK2 to oral cancer therapy.

## Data Availability

Not applicable.

## Sample Availability

Samples of the compounds are not available from the authors.

## References

[1] (2019). Cancer Registry Annual Report.

[2] Sung H., Ferlay J., Siegel R.L., Laversanne M., Soerjomataram I., Jemal A., Bray F. (2021). Global Cancer Statistics 2020: GLOBOCAN estimates of incidence and mortality worldwide for 36 cancers in 185 countries. CA Cancer J. Clin..

[3] Scott S.E., Grunfeld E.A., McGurk M. (2005). The idiosyncratic relationship between diagnostic delay and stage of oral squamous cell carcinoma. Oral Oncol..

[4] Chen Y.K., Huang H.C., Lin L.M., Lin C.C. (1999). Primary oral squamous cell carcinoma: An analysis of 703 cases in southern Taiwan. Oral Oncol..

[5] Silverman S.J. (1999). Oral cancer: Complications of therapy. Oral Surg. Oral Med. Oral Pathol. Oral Radiol. Endod..

[6] Aiguade J., Hao J., Forsyth C.J. (2001). Synthesis of a 2,9-dioxabicyclo[3.3.1]nonane via double intramolecular Hetero-Michael addition:  Entry to the F–G ring system of the azaspiracids. Org. Lett..

[7] Ganguly N.C., Mondal P., Roy S. (2013). A mild efficient iodine-catalyzed synthesis of novel anticoagulants with 2, 8-dioxabicyclo[3.3. 1]nonane core. Tetrahedron Lett..

[8] Talontsi F.M., Dittrich B., Schüffler A., Sun H., Laatsch H. (2013). Epicoccolides: Antimicrobial and antifungal polyketides from an endophytic fungus *Epicoccum* sp. associated with *Theobroma cacao*. Eur. J. Org. Chem..

[9] El Amrani M., Lai D., Debbab A., Aly A.H., Siems K., Seidel C., Schnekenburger M., Gaigneaux A., Diederich M., Feger D. (2014). Protein kinase and HDAC inhibitors from the endophytic fungus *Epicoccum nigrum*. J. Nat. Prod..

[10] Deshmukh S.K., Gupta M.K., Prakash V., Saxena S. (2018). Endophytic fungi: A source of potential antifungal compounds. J. Fungi.

[11] Chan C.K., Tsai Y.L., Chang M.Y. (2017). Construction of nitrated benzo[3.3.1]bicyclic acetal/ketal core via nitration of *o*-carbonyl allylbenzenes. Org. Lett..

[12] Nurdin L., Spasyuk D.M., Fairburn L., Piers W.E., Maron L. (2018). Oxygen-oxygen bond cleavage and formation in Co(II)-mediated stoichiometric O_2_ reduction via the potential intermediacy of a Co(IV) oxyl radical. J. Am. Chem. Soc..

[13] Li H., Li Y., Koper M.T., Calle-Vallejo F. (2014). Bond-making and breaking between carbon, nitrogen, and oxygen in electrocatalysis. J. Am. Chem. Soc..

[14] Evans C.E., Zhao Y.Y. (2017). Molecular basis of nitrative stress in the pathogenesis of pulmonary hypertension. Adv. Exp. Med. Biol..

[15] Roberts R.A., Laskin D.L., Smith C.V., Robertson F.M., Allen E.M., Doorn J.A., Slikker W. (2009). Nitrative and oxidative stress in toxicology and disease. Toxicol. Sci..

[16] Banerjee K., Ganguly A., Chakraborty P., Sarkar A., Singh S., Chatterjee M., Bhattacharya S., Choudhuri S.K. (2014). ROS and RNS induced apoptosis through p53 and iNOS mediated pathway by a dibasic hydroxamic acid molecule in leukemia cells. Eur. J. Pharm. Sci..

[17] Varga Z.V., Giricz Z., Liaudet L., Hasko G., Ferdinandy P., Pacher P. (2015). Interplay of oxidative, nitrosative/nitrative stress, inflammation, cell death and autophagy in diabetic cardiomyopathy. Biochim. Biophys. Acta.

[18] Boice A., Bouchier-Hayes L. (2020). Targeting apoptotic caspases in cancer. Biochim. Biophys. Acta Mol. Cell Res..

[19] Yeh C.C., Tseng C.N., Yang J.I., Huang H.W., Fang Y., Tang J.Y., Chang F.R., Chang H.W. (2012). Antiproliferation and induction of apoptosis in Ca9-22 oral cancer cells by ethanolic extract of *Gracilaria tenuistipitata*. Molecules.

[20] Kuo L.J., Yang L.X. (2008). Gamma-H2AX—a novel biomarker for DNA double-strand breaks. In Vivo.

[21] Omari Shekaftik S., Nasirzadeh N. (2021). 8-Hydroxy-2′-deoxyguanosine (8-OHdG) as a biomarker of oxidative DNA damage induced by occupational exposure to nanomaterials: A systematic review. Nanotoxicology.

[22] Hackenberg S., Scherzed A., Harnisch W., Froelich K., Ginzkey C., Koehler C., Hagen R., Kleinsasser N. (2012). Antitumor activity of photo-stimulated zinc oxide nanoparticles combined with paclitaxel or cisplatin in HNSCC cell lines. J. Photochem. Photobiol. B.

[23] Tang J.Y., Li L.J., Ou-Yang F., Wang C.L., Shu C.W., Wu K.H., Wang H.R., Yen C.H., Cheng Y.B., Chang H.W. (2019). Ethyl acetate extract of *Nepenthes ventricosa* × *maxima* exerts preferential killing to oral cancer cells. DNA Cell Biol..

[24] Masuelli L., Di Stefano E., Fantini M., Mattera R., Benvenuto M., Marzocchella L., Sacchetti P., Focaccetti C., Bernardini R., Tresoldi I. (2014). Resveratrol potentiates the in vitro and in vivo anti-tumoral effects of curcumin in head and neck carcinomas. Oncotarget.

[25] Freitas R.D., Dias R.B., Vidal M.T.A., Valverde L.F., Gomes Alves Costa R., Damasceno A.K.A., Sales C.B.S., Siquara da Rocha L.O., Dos Reis M.G., Soares M.B.P. (2020). Inhibition of CAL27 oral squamous carcinoma cell by targeting hedgehog pathway with vismodegib or itraconazole. Front. Oncol..

[26] Florea A.-M., Büsselberg D. (2011). Cisplatin as an anti-tumor drug: Cellular mechanisms of activity, drug resistance and induced side effects. Cancers.

[27] Cianfruglia L., Minnelli C., Laudadio E., Scire A., Armeni T. (2019). Side effects of curcumin: Epigenetic and antiproliferative implications for normal dermal fibroblast and breast cancer cells. Antioxidants.

[28] Sirichoat A., Suwannakot K., Chaisawang P., Pannangrong W., Aranarochana A., Wigmore P., Welbat J.U. (2020). Melatonin attenuates 5-fluorouracil-induced spatial memory and hippocampal neurogenesis impairment in adult rats. Life Sci..

[29] Gao X., Zhou Y., Sun H., Liu D., Zhang J., Zhang J., Liu W., Pan X. (2019). Effects of a spiroketal compound Peniciketal A and its molecular mechanisms on growth inhibition in human leukemia. Toxicol. Appl. Pharmacol..

[30] Deng Y., Zou Y., Yang C.H., Houk K.N., Smith A.B. (2021). Total syntheses of (+)-peniciketals A-B and (−)-diocollettines A exploiting a photoisomerization/cyclization union protocol. J. Org. Chem..

[31] Duong T.-H., Ha X.-P., Chavasiri W., Beniddir M.A., Genta-Jouve G., Boustie J., Chollet-Krugler M., Ferron S., Nguyen H.-H., Yamin B.M. (2018). Sanctis A–C: Three racemic procyanidin analogues from the lichen *Parmotrema sancti-angelii*. Eur. J. Org. Chem..

[32] Tang J.Y., Ou-Yang F., Hou M.F., Huang H.W., Wang H.R., Li K.T., Fayyaz S., Shu C.W., Chang H.W. (2019). Oxidative stress-modulating drugs have preferential anticancer effects—involving the regulation of apoptosis, DNA damage, endoplasmic reticulum stress, autophagy, metabolism, and migration. Semin. Cancer. Biol..

[33] Suzuki-Karasaki Y., Suzuki-Karasaki M., Uchida M., Ochiai T. (2014). Depolarization controls TRAIL-sensitization and tumor-selective killing of cancer cells: Crosstalk with ROS. Front. Oncol..

[34] Chiu C.C., Huang J.W., Chang F.R., Huang K.J., Huang H.M., Huang H.W., Chou C.K., Wu Y.C., Chang H.W. (2013). Golden berry-derived 4β-hydroxywithanolide E for selectively killing oral cancer cells by generating ROS, DNA damage, and apoptotic pathways. PLoS ONE.

[35] Huang C.H., Huang Z.W., Ho F.M., Chan W.H. (2018). Berberine impairs embryonic development in vitro and in vivo through oxidative stress-mediated apoptotic processes. Environ. Toxicol..

[36] Hung J.H., Chen C.Y., Omar H.A., Huang K.Y., Tsao C.C., Chiu C.C., Chen Y.L., Chen P.H., Teng Y.N. (2016). Reactive oxygen species mediate Terbufos-induced apoptosis in mouse testicular cell lines via the modulation of cell cycle and pro-apoptotic proteins. Environ. Toxicol..

[37] Chang H.W., Li R.N., Wang H.R., Liu J.R., Tang J.Y., Huang H.W., Chan Y.H., Yen C.Y. (2017). Withaferin A induces oxidative stress-mediated apoptosis and DNA damage in oral cancer cells. Front. Physiol..

[38] Shih H.C., El-Shazly M., Juan Y.S., Chang C.Y., Su J.H., Chen Y.C., Shih S.P., Chen H.M., Wu Y.C., Lu M.C. (2014). Cracking the cytotoxicity code: Apoptotic induction of 10-acetylirciformonin B is mediated through ROS generation and mitochondrial dysfunction. Mar. Drugs.

[39] Chien T.M., Wu K.H., Chuang Y.T., Yeh Y.C., Wang H.R., Yeh B.W., Yen C.H., Yu T.J., Wu W.J., Chang H.W. (2021). Withaferin A triggers apoptosis and DNA damage in bladder cancer J82 cells through oxidative stress. Antioxidants.

[40] Wu C.F., Lee M.G., El-Shazly M., Lai K.H., Ke S.C., Su C.W., Shih S.P., Sung P.J., Hong M.C., Wen Z.H. (2018). Isoaaptamine induces T-47D cells apoptosis and autophagy via oxidative stress. Mar. Drugs.

[41] Maione P., Gridelli C., Troiani T., Ciardiello F. (2006). Combining targeted therapies and drugs with multiple targets in the treatment of NSCLC. Oncologist.

[42] Vishwas S., Awasthi A., Corrie L., Kumar Singh S., Gulati M. (2020). Multiple target-based combination therapy of galantamine, memantine and lycopene for the possible treatment of Alzheimer’s disease. Med. Hypotheses.

[43] Chen Y.H., Hao L.J., Hung C.P., Chen J.W., Leu S.F., Huang B.M. (2014). Apoptotic effect of cisplatin and cordycepin on OC3 human oral cancer cells. Chin. J. Integr. Med..

[44] Wang S.C., Wang Y.Y., Lin L.C., Chang M.Y., Yuan S.F., Tang J.Y., Chang H.W. (2020). Combined treatment of sulfonyl chromen-4-ones (CHW09) and ultraviolet-C (UVC) enhances proliferation inhibition, apoptosis, oxidative stress, and DNA damage against oral cancer cells. Int. J. Mol. Sci..

[45] Tang J.Y., Shu C.W., Wang C.L., Wang S.C., Chang M.Y., Lin L.C., Chang H.W. (2019). Sulfonyl chromen-4-ones (CHW09) shows an additive effect to inhibit cell growth of X-ray irradiated oral cancer cells, involving apoptosis and ROS generation. Int. J. Radiat. Biol..

[46] Ekins S., Andreyev S., Ryabov A., Kirillov E., Rakhmatulin E.A., Sorokina S., Bugrim A., Nikolskaya T. (2006). A combined approach to drug metabolism and toxicity assessment. Drug Metab Dispos.

[47] Ekins S., Nikolsky Y., Bugrim A., Kirillov E., Nikolskaya T. (2007). Pathway mapping tools for analysis of high content data. Methods Mol. Biol.

[48] Foyer C.H., Noctor G. (2005). Redox homeostasis and antioxidant signaling: A metabolic interface between stress perception and physiological responses. Plant Cell.

[49] Willems P.H., Rossignol R., Dieteren C.E., Murphy M.P., Koopman W.J. (2015). Redox homeostasis and mitochondrial dynamics. Cell Metab..

[50] Peng S.Y., Lin L.C., Chen S.R., Farooqi A.A., Cheng Y.B., Tang J.Y., Chang H.W. (2021). Pomegranate extract (POMx) induces mitochondrial dysfunction and apoptosis of oral cancer cells. Antioxidants.

[51] Yang C.Y., Meng C.L. (1994). Regulation of PG synthase by EGF and PDGF in human oral, breast, stomach, and fibrosarcoma cancer cell lines. J. Dent. Res..

[52] Chang Y.T., Huang C.Y., Li K.T., Li R.N., Liaw C.C., Wu S.H., Liu J.R., Sheu J.H., Chang H.W. (2016). Sinuleptolide inhibits proliferation of oral cancer Ca9-22 cells involving apoptosis, oxidative stress, and DNA damage. Arch. Oral Biol..

[53] Vignon C., Debeissat C., Georget M.T., Bouscary D., Gyan E., Rosset P., Herault O. (2013). Flow cytometric quantification of all phases of the cell cycle and apoptosis in a two-color fluorescence plot. PLoS ONE.

[54] Lee C.H., Shih Y.L., Lee M.H., Au M.K., Chen Y.L., Lu H.F., Chung J.G. (2017). Bufalin induces apoptosis of human osteosarcoma U-2 OS cells through endoplasmic reticulum stress, caspase- and mitochondria-dependent signaling pathways. Molecules.

[55] Liu S.L., Yang K.H., Yang C.W., Lee M.Y., Chuang Y.T., Chen Y.N., Chang F.R., Chen C.Y., Chang H.W. (2021). Burmannic acid inhibits proliferation and induces oxidative stress response of oral cancer cells. Antioxidants.

